# Protein glycation products associate with progression of kidney disease and incident cardiovascular events in individuals with type 1 diabetes

**DOI:** 10.1186/s12933-024-02316-w

**Published:** 2024-07-04

**Authors:** Krishna Adeshara, Daniel Gordin, Anni A. Antikainen, Valma Harjutsalo, Niina Sandholm, Markku J. Lehto, Per-Henrik Groop, on behalf of the FinnDiane Study Group

**Affiliations:** 1 Folkhälsan Research Center, Helsinki, Finland; 2 Research Program for Clinical and Molecular Metabolism, Faculty of Medicine, University of Helsinki, Helsinki, Finland; 3 Department of Nephrology, University of Helsinki and Helsinki University Hospital, Helsinki, Finland; 4 Joslin Diabetes Center, Harvard Medical School, Boston, MA USA; 5 Minerva Foundation Institute for Medical Research, Helsinki, Finland; 6 Department of Diabetes, Central Clinical School, Monash University, Melbourne, VIC Australia; 7 FRCPE Folkhälsan Research Center, Biomedicum Helsinki, University of Helsinki, Haartmaninkatu 8, PO Box 63, 00290 Helsinki, Finland

**Keywords:** Fructosamine, Advanced glycation end products, MG-H1, Type 1 diabetes, Diabetic kidney disease, MACE

## Abstract

**Background:**

Despite improved glycemic treatment, the impact of glycation on pathological consequences may persist and contribute to adverse clinical outcomes in diabetes. In the present study we investigated the association between serum protein glycation products and progression of kidney disease as well as incident major adverse cardiovascular events (MACE) in type 1 diabetes.

**Methods:**

Fructosamine, advanced glycation end products (AGEs), and methylglyoxal-modified hydro-imidazolone (MG-H1) were measured from baseline serum samples in the FinnDiane study (*n* = 575). Kidney disease progression was defined as steep eGFR decline (> 3 mL/min/1.73 m^2^/year) or progression of albuminuria (from lower to higher stage of albuminuria). MACE was defined as acute myocardial infarction, coronary revascularization, cerebrovascular event (stroke), and cardiovascular death.

**Results:**

Fructosamine was independently associated with steep eGFR decline (OR 2.15 [95% CI 1.16–4.01], *p* = 0.016) in the fully adjusted model (age, sex, baseline eGFR). AGEs were associated with steep eGFR decline (OR 1.58 per 1 unit of SD [95% CI 1.07–2.32], *p* = 0.02), progression to end-stage kidney disease (ESKD) (HR 2.09 per 1 unit of SD [95% CI 1.43–3.05], *p* < 0.001), and pooled progression (to any stage of albuminuria) (HR 2.72 per 1 unit of SD [95% CI 2.04–3.62], *p* < 0.001). AGEs (HR 1.57 per 1 unit of SD [95% CI 1.23–2.00], *p* < 0.001) and MG-H1 (HR 4.99 [95% CI 0.98–25.55], *p* = 0.054) were associated with incident MACE. MG-H1 was also associated with pooled progression (HR 4.19 [95% CI 1.11–15.89], *p* = 0.035). Most AGEs and MG-H1 associations were no more significant after adjusting for baseline eGFR.

**Conclusions:**

Overall, these findings suggest that protein glycation products are an important risk factor for target organ damage in type 1 diabetes. The data provide further support to investigate a potential causal role of serum protein glycation in the progression of diabetes complications.

**Supplementary Information:**

The online version contains supplementary material available at 10.1186/s12933-024-02316-w.

## Introduction

Diabetic kidney disease (DKD) is the most common cause of chronic kidney disease (CKD) affecting 40% of individuals with diabetes and is associated with high risk of cardiovascular mortality [1, 2]. Chronic hyperglycemia is associated with the risk of DKD, characterized by progressive albuminuria and declining rate of estimated glomerular filtration rate (eGFR). Despite improved glycemic treatment, there is still a substantial residual risk of DKD in individuals with type 1 diabetes [3]. There may be additional factors, which have not improved upon intensive treatments and may therefore contribute to long-lasting pathological damage in diabetes.

Glycation products are heterogeneous molecules formed through non-enzymatic reactions between reducing sugars and proteins, lipids, or nucleic acids. Non-enzymatic glycation is a multi-step reaction where an early glycation product, fructosamine, reflects total glycated blood proteins as well as medium-term glycemia, and contributes to the formation of irreversible advanced glycation end products (AGEs). These unstable heterogeneous AGEs can accumulate or crosslink with proteins and thus harm cellular functional activities such as the redox balance, apoptosis, or vascular stiffening [4]. Methylglyoxal is a highly reactive dicarbonyl metabolite, mainly formed inside the cell and acts as a potent precursor of the AGEs. Partial leakage of methylglyoxal from a cell can impair the activity of matrix protein collagen IV and induce endothelial cell detachment [5]. Protein glycation products are divergent in their structures, and dependent on the kidneys for their excretion and reabsorption. Kidney dysfunction and declining eGFR over time increase the accumulation of AGEs in plasma [6, 7, 9], which may reflect progressive kidney damage in diabetes.

Protein glycation products have long been considered predictors of risk of micro- and macrovascular complications in diabetes. Previous studies have shown increased plasma concentrations of AGEs in individuals on hemodialysis [6], as well as in individuals with type 1 diabetes with reduced eGFR [8]. Accumulation of methylglyoxal-modified hydro-imidazolone (MG-H1) was increased by ninefold in individuals on peritoneal dialysis compared to healthy controls [9]. AGE-modified LDL-cholesterol [10] and skin autofluorescence of AGEs were associated with atherosclerosis in the elderly [11] and in a diabetes population [12], and progression of kidney disease in type 1 diabetes [13]. Though these observations reflect the strong association of glycation products with the development of kidney disease, there are several aspects that remain unclear, e.g., (1) does accumulated glycation products directly contribute to kidney disease or are they just a mere marker of metabolic disturbances; (2) are currently available data enough to support the clinical utility of glycation products as a marker of early changes related to diabetes complications in clinical practice; (3) do glycation products increase long-term risk of adverse clinical outcomes. Additionally, pre-clinical and clinical trials have generated mixed results about the effect of glycation products on related pathologies [10, 13, 14], and longitudinal studies with longer follow-up are required to expand the understanding of glycation-mediated kidney damage.

In the present study, we assessed the associations of different serum protein glycation products such as the early glycation product fructosamine, AGEs representing later stage end-products, and dicarbonyl (methylglyoxal) modified glycation products (MG-H1), with the progression of DKD defined either as steep eGFR decline or progression of albuminuria in individuals with type 1 diabetes. Furthermore, we aimed to evaluate the associations of these different glycation products with the risk of incident major adverse cardiovascular events (MACE) in type 1 diabetes.

## Research design and methods

### Study participants

This study included 575 adult participants from the Finnish Diabetic Nephropathy (FinnDiane) Study, a nationwide longitudinal multicenter study. At baseline, participants were grouped into (1) non-diabetic (*n* = 128); (2) type 1 diabetes (*n* = 329) without DKD; (3) type 1 diabetes with DKD (*n* = 80); or (4) type 1 diabetes with end stage kidney disease (ESKD, *n* = 38). All individuals with ESKD had undergone dialysis, and 16 of them had received a subsequent kidney transplant.

### Clinical information

Type 1 diabetes was defined as age at diabetes onset < 40 years and permanent insulin treatment started within 1 year after the diagnosis. The study protocol is in accordance with the Declaration of Helsinki and approved by the Ethics Committee of the Helsinki and Uusimaa Hospital District (HUS) (491/E5/2006, 238/13/03/00/2015, and HUS-3313-2018, July 3rd 2019). All participants gave their informed written consent.

### Data on albuminuria status

Urinary albumin excretion rate (AER) was determined at the FinnDiane baseline and follow-up visits from at least two timed overnight or 24-h urine collections. Normal AER was defined as AER < 20 µg/min or < 30 mg/24 h, moderate albuminuria as AER ≥ 20 and < 200 µg/min or ≥ 30 and < 300 mg/24 h, severe albuminuria as AER ≥ 200 µg/min or ≥ 300 mg/24 h, and ESKD as being on dialysis or having received a kidney transplant. DKD was defined as the presence of moderate or severe albuminuria.

The data on albuminuria progression were collected from all available medical records and health care registries until the end of year 2019. Progression of albuminuria was defined as progression from a lower stage to any higher stage of albuminuria [normal AER to moderate albuminuria; moderate to severe albuminuria; severe albuminuria to ESKD]. From baseline to the end of the follow-up time (median of 11 years), nine individuals progressed form normal AER to moderate albuminuria, five individuals progressed from moderate albuminuria to severe albuminuria, and 17 individuals progressed from severe albuminuria to ESKD. Early DKD progression comprised those, who progressed from normal AER to moderate albuminuria, or from moderate to severe albuminuria (*n* = 14); and pooled progression comprised progression to any stage of albuminuria (normal AER to moderate albuminuria, moderate to severe albuminuria, severe albuminuria to ESKD; *n* = 31). Individuals with baseline ESKD were excluded from the analysis when assessing the association of glycation products with albuminuria progression. Albuminuria progression refers to worsening of albuminuria over time and risk of ESKD, whereas progression to ESKD reflects severely impaired kidney function.

### Data on estimated glomerular filtration rate (eGFR)

eGFR was calculated using the Chronic Kidney Disease Epidemiology Collaboration equation [15] based on serum creatinine measurements at the study baseline and follow-up visits. The decline of eGFR was calculated by subtracting the last eGFR measurement from the baseline measurement and dividing it by the time between the measurements. A steep decline was defined by an average loss of eGFR more than 3 mL/min/1.73 m^2^/year, and 45 individuals experienced steep eGFR decline over the median follow-up of 11 years.

### Data on major adverse cardiovascular events (MACE)

Data on cardiovascular status were registered by a standardized questionnaire, which was completed by the individual’s attending physician and verified from the medical files until the year 2017. MACE was defined as acute myocardial infraction, coronary revascularization, cerebrovascular event (stroke), and cardiovascular death. A total of 20 individuals had developed incident MACE over the median follow-up of 11 years, where four had suffered a stroke, eight a coronary event, one had both a stroke and a coronary event, and seven individuals had died. The cause of death was unknown in three individuals, which were however included as MACE cases.

### Analysis of different glycation products

We have measured three different serum glycation products from serum collected at the study baseline visit and stored in − 80 °C. Serum fructosamine was measured by a colorimetric technique [16] and the absorbance was measured at 540 nm. Fructosamine content was calculated using standard 1-deoxy-1 morpholino-d-fructose (0–3.2 mM/L). Serum AGEs were determined with an in-house fluorometric assay as described earlier [17], which measures different AGE modifications (e.g., crossline, fluorolink, pyrropyridine and vesperlysine). The measured fluorescence was not an AGE itself, but it rather indirectly indicates the amount of AGEs modification present in the sample. Fluorescence intensity was measured at emission wavelength 460 nm upon excitation wavelength 360 nm using a multi-mode microplate reader (Synergy 2, BioTek, Potton, United Kingdom) at room temperature. The mean of duplicated readings was calculated and presented in arbitrary units (AU). Serum MG-H1 protein adducts were measured by competitive ELISA (STA-811-5, Cell Biolabs, San Diego, USA) according to the manufacturers’ instructions. Absorbance was taken at 450 nm, and the quantity of MG-H1 was determined by comparing with a known MG-BSA standard.

### Statistical analysis

Descriptive clinical characteristics are presented as percentages for categorical variables, median (interquartile range) for non-normally distributed continuous variables and mean ± SD for continuous variables with normal distribution. Differences in protein glycation products between the groups were assessed by Mann–Whitney *U* test or Kruskal–Wallis test. AGEs were standardized by subtracting with population mean and dividing by the population standard deviation (SD). Log-transformation was used to obtain normal distribution for MG-H1. Subsequently, univariable, and binary logistic regression was performed to assess the influence of circulatory protein glycation products on probabilities for steep eGFR decline. Cox regression analysis was performed to assess the association of protein glycation products with the risk of progression of complications in type 1 diabetes. Cox regression models were adjusted for age, sex, LDL-cholesterol, and baseline eGFR, when applicable. Furthermore, to study the effect of HbA1c on observed associations between protein glycation products and clinical outcomes, Cox regression was performed by additionally adjusting for HbA1c. The cumulative survival is illustrated using Kaplan–Meier curves and probabilities compared with the log-rank test. Apart from the diabetes vs. non-diabetes group comparisons shown in Table 1 and Supplementary Fig. 1, all subsequent analyses on progression of kidney disease or incident MACE were performed uniquely in individuals with type 1 diabetes. All analyses were performed using IBM SPSS Statistics 26.0 (IBM Corporation, Somers, NY, USA). Univariable logistic regression curves were made with the SAS version 9.2 software (SAS Institute Inc., Cary, NC).

**Table 1:** Baseline characteristics of participants according to the status of diabetic kidney disease

Phenotype	ND (*n* = 128)	T1D non-DKD (*n* = 329)	T1D DKD (*n* = 80)	T1D ESKD (*n* = 38)
Men, n (%)	89 (70)	127 (39)	43 (54)	27 (71)
Age, years	42.7 ± 12.7	33.6 ± 10.3^a1^	44.2 ± 12.5^b1^	43.8 ± 8.9 ^b1^
Diabetes duration, years	–	16.8 ± 11.5	30.9 ± 11.5^b1^	33.2 ± 9.1 ^b1^
Systolic blood pressure, mmHg	130.9 ± 15.2	127.6 ± 14.6^a3^	143.2 ± 20.8^a1, b1^	151.7 ± 22.9^a1, b1^
Diastolic blood pressure, mmHg	78.9 ± 10.3	75.9 ± 8.7^a2^	79.0 ± 11.2^b3^	83.8 ± 14.8^a3, b1^
HbA_1c_, %	5.3 ± 0.28	7.8 ± 1.23^a1^	8.4 ± 1.25^a1, b1^	8.1 ± 1.46^a1^
HbA_1c_, mmol/mol	33.9 ± 3.0	62.2 ± 13.5^a1^	68.6 ± 13.8^a1, b1^	64.5 ± 16.0^a1^
Total cholesterol, mmol/L	4.9 ± 0.97	4.6 ± 0.81^a1^	4.6 ± 1.13^a2^	4.2 ± 1.07^a1, b2^
HDL-cholesterol, mmol/L	1.3 ± 0.39	1.5 ± 0.39^a1^	1.5 ± 0.48^a2^	1.4 ± 0.53^b3^
LDL-cholesterol, mmol/L	3.4 ± 0.98	2.7 ± 0.72^a1^	2.8 ± 1.00^a1^	2.4 ± 0.89^a1, b3^
Triglycerides, mmol/L	1.2 ± 0.73	1.1 ± 0.78	1.3 ± 0.67^b2^	1.7 ± 0.78^a1, b1^
eGFR, mL/min/1.73 m^2^	96.3 (84.5–109.7)	109.6 (99.7-118.5)^a1^	85.3 (35.6-105.1)^a1, b1^	11.7 (8.8–53.8)^a1, b1^
eGFR < 60 mL/min/1.73 m², n (%)	NA	2 (0.6)	24 (30)	30 (79)
Fructosamine, mM/L	1.47 ± 0.37	1.87 ± 0.47^a1^	1.90 ± 0.52^a1^	1.76 ± 0.21^a1^
AGEs, AU	11,397 (10,001–12,971)	10,670 (9159–12,310)^a2^	17,575 (11,171–21,647)^a1, b1^	35,201 (21,905–41,524)^a1, b1^
MG-H1, µg/ml	2.10 (1.65–2.47)	2.29 (1.60–3.08)	1.79 (1.53–2.65)	3.29 (2.06–7.11)^a1, b1^

Data are shown as percentages for categorical variables, median (interquartile range) for nonnormally distributed continuous variables, and mean ± SD for continuous variables with normal distribution. Between-group comparisons were done by Mann–Whitney *U* test. The T1D non-DKD group includes individuals with normal AER. T1D DKD group includes individuals with moderate and severe albuminuria

^a^compared with non-diabetic group, b: compared with non-DKD T1D group. ^a1, b1,^ = ***(*p* < 0.001); ^a2, b2^ = **(*p* < 0.01); ^a3, b3^ = *(*p* < 0.05)

HbA_1c_, glycated hemoglobin A_1c_; HDL-cholesterol, high-density lipoprotein cholesterol; LDL-cholesterol, low-density lipoprotein cholesterol; eGFR, estimated glomerular filtration rate; AGEs, advanced glycation end products; MG-H1, methylglyoxal-modified hydro-imidazolone; AER, albumin excretion rate; NA, not applicable

### Data and resource availability

Individual-level data of the study participants are not publicly available because of the restrictions due to the study consent provided by the participant at the time of data collection.

## Results

### Clinical characteristics of the study subjects

The individuals with type 1 diabetes were stratified based on their albuminuria status into non-DKD, DKD, and ESKD groups. Individuals in the non-DKD type 1 diabetes group were younger with lower blood pressure (BP), increased HbA1c, and higher eGFR compared to the non-diabetic controls. The individuals with DKD and ESKD were older and had longer diabetes duration than the non-DKD group. BP was increased in the DKD and ESKD groups, and HbA1c was higher in DKD compared to both the non-diabetic controls and the non-DKD group. Total and LDL-cholesterol were lower in all three type 1 diabetes groups compared to the non-diabetic controls. The eGFR decreased with worsening DKD status and being the lowest in the ESKD group (Table 1).

### Levels of protein glycation products as per albuminuria and eGFR

In cross-sectional analysis, fructosamine was elevated in the non-DKD, DKD, and ESKD groups compared to the non-diabetic controls (*p* < 0.001), however, no difference was observed between the non-DKD and DKD groups. AGEs were moderately reduced (*p* < 0.01) in non-DKD compared to non-diabetic controls, whereas further elevated in the DKD (*p* < 0.001) and ESKD groups (*p* < 0.001) compared to both non-diabetic controls and non-DKD group. MG-H1 was higher in the ESKD group compared to both non-diabetic controls and the non-DKD group (*p* < 0.001), however, no difference was observed between the non-DKD and the DKD group (Table 1 and Supplementary Fig. 1A–C). MG-H1 (*p* < 0.001) was lower in those with a kidney transplant compared to those on dialysis, while no difference was observed in fructosamine and AGEs between the dialysis and kidney transplant groups (Supplementary Fig. 1D–F). When participants were stratified based on eGFR as per KDIGO classifications [18], the AGEs increased with lower eGFR across the groups (*p* < 0.001). MG-H1 also varied across the groups (*p* < 0.001) being the highest in those with eGFR < 15 ml/min/1.73m^2^ (Supplementary Fig. 1G–I).

### Protein glycation products and progression of kidney disease

In longitudinal analysis, the univariable analysis showed increased probability for steep eGFR decline by increasing levels of fructosamine and AGEs (Supplementary Fig. 2A, B). This was further confirmed in binary logistic regression analysis where each 1 unit increase in fructosamine independently increased the odds of steep eGFR decline (model 3, adjusted for age, sex, and baseline eGFR: OR 2.15 [95% CI 1.16–4.01], *p* = 0.016; Table 2). As an additional sensitivity analysis, this association remained significant (OR 2.02 [95% CI 1.07–3.79], *p* = 0.03) even after additionally adjusting for HbA1c, suggesting that fructosamine is not merely a marker of hyperglycemia. Additionally, 1 SD increase in standardized AGEs increased the odds of steep eGFR decline (OR 1.58 per 1 unit of SD [95% CI 1.07–2.32], *p* = 0.02) when adjusted for age and sex but not after adjustment for baseline eGFR. MG-H1 did not show any association with steep eGFR decline (Table 2).

**Table 2:** Binary logistic regression of protein glycation products with steep eGFR decline

	OR1 (95% CI)	*P*-value	OR2 (95% CI)	*P*-value	OR3 (95% CI)	*P*-value
Fructosamine	1.87 (1.03, 3.40)	**0.041**	2.23 (1.19, 4.13)	**0.011**	2.15 (1.16, 4.01)	**0.016**
AGEs	1.88 (1.31, 2.69)	**< 0.001**	1.58 (1.07, 2.32)	**0.020**	1.53 (0.82, 2.85)	0.180
MG-H1	0.63 (0.17, 2.31)	0.490	0.57 (0.15, 2.13)	0.403	0.43 (0.11, 1.74)	0.237

Bold numbers: represent significant p-values

A total of 306 patients had available follow-up data and in general 45 individuals had developed steep eGFR decline (FA analysis: 45 steep decline vs. 261 controls; AGEs analysis: 43 steep decline vs. 257 controls; and MG-H1 analysis: 45 steep decline vs. 261 controls)

To obtain normal distribution MG-H1 was log-transformed

OR1, unadjusted; OR2, adjusted for age and sex; OR3, adjusted for age, sex, and baseline eGFR

AGEs, advanced glycation end products; MG-H1, methylglyoxal-modified hydro-imidazolone

In Cox regression, AGEs were associated with progression from severe albuminuria to ESKD (HR 2.09 per 1 unit of SD [95% CI 1.43–3.05], *p* < 0.001) and with pooled progression (HR 2.72 per 1 unit of SD [95% CI 2.04–3.62], *p* < 0.001) when adjusted for age and sex, but not after adjustment for baseline eGFR (Table 3). AGEs were also associated with early progression to DKD in the unadjusted model (HR 3.62 per 1 unit of SD [95% CI 1.12–11.71], *p* = 0.03). Additionally, log-transformed MG-H1 was associated with pooled progression (HR 4.19 [95% CI 1.11–15.89], *p* = 0.035) when adjusted for age and sex but not after adjustment for baseline eGFR. Fructosamine did not show any significant association with progression of albuminuria (Table 3).

**Table 3:** Cox regression analysis of association of protein glycation products with progression of albuminuria and incident major adverse cardiovascular events

	Phenotype	*N* cases/controls	Model 1		Model 2		Model 3	
			HR1 (95% CI)	*P*-value	HR2 (95% CI)	*P*-value	HR3 (95% CI)	*P*-value
Fructosamine	DKD (pooled early progression)	14/253	0.47 (0.07, 2.95)	0.417	0.60 (0.09, 3.81)	0.592	0.62 (0.11, 3.53)	0.588
	Severe AER to ESKD	17/19	0.85 (0.26, 2.74)	0.786	0.82 (0.23, 2.95)	0.759	1.08 (0.15, 8.05)	0.941
	Pooled progression	31/278	0.59 (0.20, 1.71)	0.327	0.92 (0.32, 2.60)	0.873	0.76 (0.23, 2.51)	0.652
AGEs	DKD (pooled early progression)	14/ 253	3.62 (1.12, 11.71)	**0.032**	2.95 (0.86, 10.11)	0.086	2.29 (0.59, 8.98)	0.232
	Severe AER to ESKD	17/19	2.08 (1.44, 3.00)	**< 0.001**	2.09 (1.43, 3.05)	**< 0.001**	1.00 (0.55, 1.82)	0.999
	Pooled progression	31/278	3.20 (2.48, 4.12)	**< 0.001**	2.72 (2.04, 3.62)	**< 0.001**	1.12 (0.71, 1.79)	0.622
MG-H1	DKD (pooled early progression)	14/253	2.50 (0.32, 19.41)	0.381	2.29 (0.30, 17.42)	0.421	2.39 (0.29, 19.83)	0.420
	Severe AER to ESKD	17/19	3.37 (0.73, 15.53)	0.119	3.49 (0.50, 24.14)	0.206	0.18 (0.01, 3.04)	0.233
	Pooled progression	31/278	4.15 (1.14, 15.11)	**0.031**	4.19 (1.11, 15.89)	**0.035**	1.26 (0.26, 6.07)	0.771
Fructosamine	Incident MACE^†^	20/304	0.68 (0.19, 2.42)	0.555	1.56 (0.51, 4.78)	0.437	1.30 (0.39, 4.39)	0.668
AGEs	Incident MACE^†^	20/304	1.83 (1.48, 2.25)	**< 0.001**	1.57 (1.23, 2.00)	**< 0.001**	1.02 (0.67, 1.58)	0.913
MG-H1	Incident MACE^†^	20/304	4.47 (1.00, 19.94)	**0.049**	4.99 (0.98, 25.55)	**0.054**	2.79 (0.43, 18.14)	0.282

Bold numbers: represent significant p-values

DKD (pooled early progression) comprises those who progressed from normal AER to moderate albuminuria, *n* = 9; or from moderate to severe albuminuria, *n* = 5)

17 individuals progressed from severe albuminuria to ESKD

Pooled progression comprised 31 individuals who progressed to any stage of albuminuria (normal AER to moderate albuminuria, *n* = 9; moderate to severe albuminuria, *n* = 5; severe albuminuria to ESKD, *n* = 17)

Model 1: unadjusted

Model 2: adjusted for age and sex; ^†^adjusted for age, sex and LDL-cholesterol

Model 3: adjusted for age, sex and baseline eGFR; ^†^adjusted for age, sex, LDL-cholesterol, and baseline eGFR

To obtain normal distribution MG-H1 was log-transformed

MACE, major adverse cardiovascular events; AGEs, advanced glycation end products; MG-H1, methylglyoxal-modified hydro-imidazolone

### Protein glycation products and incident MACE

AGEs (HR 1.57 per 1 unit of SD [95% CI 1.23–2.00], *p* < 0.001) and log-transformed MG-H1 (HR 4.99 [95% CI 0.98–25.55], *p* = 0.054) were associated with incident MACE when adjusted for age, sex, and LDL-cholesterol but not after adjustment for baseline eGFR. Fructosamine did not show any significant associations with incident MACE (Table 3).

**Fig. 1:**
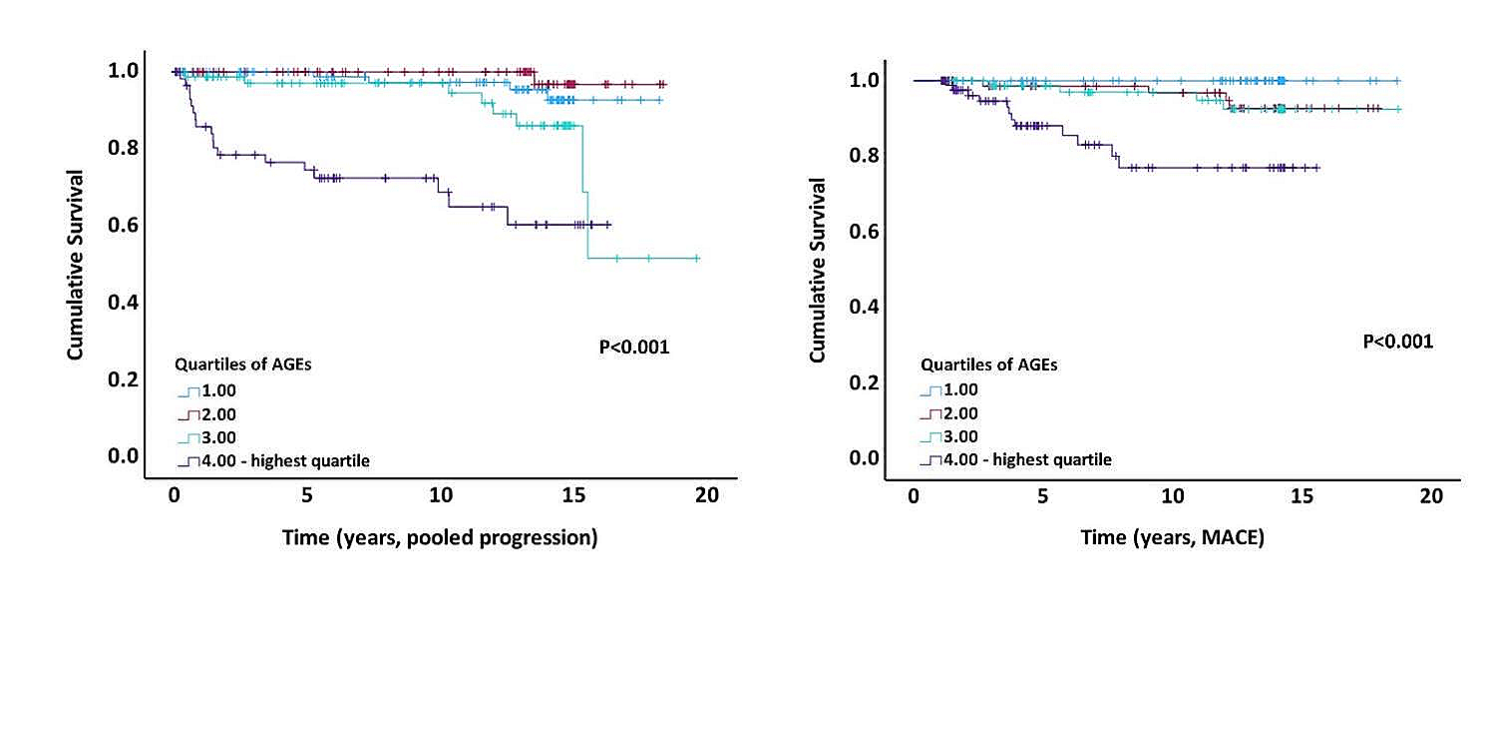
Kaplan–Meier plot for progression of diabetes kidney disease (**A**), and incident major adverse cardiovascular event (MACE; **B**). Individuals were stratified by quartiles of advanced glycation end products (AGEs) at baseline

As shown in the survival curves in Fig. 1 both for kidney disease progression and incident MACE, the risk of an event differed when compared across the AGE quartiles (*p* < 0.001, Fig. 1A, B), being particularly pronounced for individuals in the highest quartile (Q4) for AGEs: at 10-year follow-up, individuals in the top quartile of AGEs had 65% survival without albuminuria progression, compared to those in the third quartile with 95% survival without albuminuria progression. Similarly, for the 10-year cumulative risk of incident MACE, individuals in the top quartile of AGEs had 77% survival without MACE when compared to those in the third quartile with 97% survival without MACE. Of note, there were no incident MACE events observed in the individuals in the lowest quartile of AGEs (Q1).

## Discussion

In this work we have conducted a comprehensive study of three different protein glycation products, representing different stages of non-enzymatic glycation processes, and their potential role for DKD and incident MACE in individuals with type 1 diabetes, both cross-sectionally and prospectively with a median follow-up of 11 years. Fructosamine did not differ between the DKD groups in the cross-sectional analysis but served as an independent risk factor of rapid eGFR decline even after adjustment for well-known risk factors, such as eGFR and HbA1c. AGEs were elevated in the DKD and ESKD groups, and prospectively associated with rapid eGFR decline, progression of albuminuria, and incident MACE. The increased risk was observed among those individuals in the top quartile of AGEs. Dicarbonyl-derived MG-H1 was also significantly associated with the progression of albuminuria and moderately associated with incident MACE. However, the AGEs and MG-H1 associations did not remain significant after adjusting for baseline eGFR.

Glycation products can be formed through different pathways and through different precursors, and their circulatory levels are therefore affected by various factors including clinical and demographic characteristics. Glycation of proteins is a natural process continuously occurring in the circulatory systems and remains high in manifest diabetes [9]. Fructosamine reflects medium-term glycemic control, and elevated levels have been shown to associate with micro- or macrovascular complications and mortality in individuals with diabetes [19] and in non-diabetic subjects [20] in cross-sectional analyses. Fructosamine was also associated with increased risk of all-cause mortality and first sepsis hospitalization in 100 individuals with diabetes on hemodialysis during a prospective 3-year follow-up [21]. Mild elevation of serum fructosamine was associated with decreased eGFR in non-diabetic individuals without CKD over a median follow-up of 3.5 years [22]. Unlike the late glycation products, fructosamine may capture recent glycemic excursions more closely which makes it more capable of identifying acute changes associated with kidney disease. Our study extends these findings and investigated the longitudinal associations in type 1 diabetes, where fructosamine was associated with a 2.15-fold increased risk of steep eGFR decline. However, our study demonstrated no association of fructosamine with the progression of albuminuria. Of note, fructosamine concentration not only depends on glucose levels but is largely affected by the serum protein concentration, metabolism, and protein turnover as it constitutes to a large extent of glycated albumin (80%), glycated lipoproteins, and globulins. It is possible that the individuals with kidney disease in the present study might have low in situ rate of glycation of serum proteins due to less availability of albumin, or these individuals might excrete more serum proteins in the urine due to their kidney dysfunction. If true, this might explain why we observed no association between fructosamine and progression of albuminuria. Nevertheless, the robust association between fructosamine and steep eGFR decline suggests that fructosamine could serve as a potential early diagnostic marker to predict change in kidney function. Additionally, fructosamine’s ability to reflect short-term glycemic changes makes it a more sensitive marker to capture recent glycemic excursions and its effect on kidney function or CKD progression.

Serum AGEs have widespread effects on protein damage in multiple systems by altering structural quality of blood vessels, bones, and other tissues through protein cross-linking [9]. AGEs are mainly excreted via the kidneys and their circulatory levels are, therefore, highly dependent on kidney function. Impaired glomerular function or reduced eGFR have been linked to increased accumulation of glycated products in the circulation. Accumulated glycated products can cause glomerular basement membrane thickening, mesangial expansion, podocyte injury, compromising the filtration barrier and reducing glomerular filtration rate. Declining of kidney function and reduced eGFR reflects impaired clearance of AGEs from the circulation. A previous study showed serum accumulation of AGEs in children with chronic kidney failure and type 1 diabetes [23]. In line, the present study reports elevated serum AGEs with worsening kidney status in type 1 diabetes. However, elevated serum AGEs are also known to have various detrimental effects on kidney health. AGEs work synergistically with other pathways including oxidative stress, hypertension, or the renin angiotensin-aldosterone system, and promotes progressive kidney injury likely via fibrogenesis, phenotypic differentiation or cell death. Furthermore, AGEs form molecular cross-links with extracellular matrix (ECM) proteins, increase expansion of the ECM area, and affects tissue remodeling, which is an important signature of progression of CKD [9, 24]. The present study supports such findings and reports an association of AGEs with measures of worsening kidney function (steep eGFR decline and progression to albuminuria) in type 1 diabetes in a prospective setting.

In addition, circulating AGEs have been associated with vascular stiffening in individuals with type 2 diabetes [25], the degree of coronary arteriosclerosis in non-diabetic subjects [26], impaired ventricular function in individuals with type 1 diabetes [27], and AGE-receptor mediated endothelial dysfunction in individuals with CKD partly explaining cardiovascular mortality [28]. A recent study by Koska et al. [29] reported an association between elevated AGEs and incident CVD in type 2 diabetes. The present study extends these findings to individuals with type 1 diabetes and demonstrates an association of AGEs with incident MACE.

Methylglyoxal is a highly reactive dicarbonyl compound produced through metabolic flux at intracellular concentrations of 1–4 µM and a half-life of 2–4 h. Methylglyoxal interacts with arginine residues of proteins to form MG-H1, a dominant hydroimidazolone glycation product found in both serum and tissues. Methylglyoxal was shown to impair the ability of the kidney to remove damaged proteins through covalent modification of the proteasome, and elevated expression of mRNA of pro-inflammatory and oxidative stress pathways in the kidney transcriptome [30]. Both methylglyoxal and methylglyoxal-modified proteins have been associated with the pathology of multiple diseases including diabetes, cancer, liver, and kidney diseases. The experimental glyoxalase 1 knockout mice showed elevated MG-H1 adducts in glomeruli and tubules with the development of albuminuria and mesangial expansion in a non-diabetic condition [31]. The present study extends these findings and reports an association of MG-H1 with increased risk of progression of albuminuria. Previous studies showed an association of plasma free methylglyoxal with incident CVD in both type 1 and type 2 diabetes [32, 33]. Subsequently, accumulation of methylglyoxal on vascular endothelial cells increases redox imbalance, vascular resistance, insulin resistance, salt sensitivity and retention of body fluid volume [34, 35]. Methylglyoxal has been shown to be a predictor of intima-media thickening, vascular stiffening, and hypertension over 5-years of follow-up in individuals with type 2 diabetes [36]. Interestingly, postprandial hyperglycemia induced increased intracellular concentration of methylglyoxal, which has been linked to the development of macroangiopathy in individuals with type 2 diabetes [37]. A recently published study by Nakamura et al. [38] showed lower risk of combined cardiovascular events in people with type 2 diabetes with continuous low levels of MG-H1. The present study extended these findings to individuals with type 1 diabetes and demonstrates the moderate association of protein bound MG-H1 with incident MACE in type 1 diabetes. Interestingly, MG-H1 was elevated only in those with ESKD, and no difference was observed in DKD and non-DKD groups compared to non-diabetic controls. Similarly, Perkins et al. [39] showed that the plasma protein bound MG-H1 content did not differ between those with stable eGFR and those with early eGFR decline in individuals with type 1 diabetes and moderate albuminuria. However, the fractional urinary excretion of MG-H1 free adducts was increased 100% in those with early eGFR decline. The reduced MG-H1 levels in those after kidney transplantation also suggest the possibility of a kidney dependent alteration in MG-H1 levels in the present study. These findings warrant analysis of fractional excretion of this analyte in individuals with kidney disease.

The main limitation of this study is the relatively small sample size, although this is among the largest prospective studies. Furthermore, we measured only circulatory protein bound glycation products but not free glycated adduct residues or tissue AGEs. We did not have data available on dietary AGEs. All serum AGEs do not exhibit fluorescence, therefore, some non-fluorescent AGEs might not have been detected with the quantitative detection method of AGEs. However, we believe that these limitations do not reduce the importance of the reported associations as if anything they would have diluted our findings. The study also has some strengths including well-characterized Finnish participants with type 1 diabetes, prospective study design with follow-up visits, data on CVD events, kidney disease as well as high quality and completeness of national registry data. In addition, we had access to registry data including all individuals with type 1 diabetes, who had received a kidney transplant from the beginning of transplant activities in Finland. We also have access to death certificates and only 8% had an undetermined cause of death which provides a comprehensive picture of the comorbidities that affect the patient survival.

In conclusion, fructosamine is an independent predictor of steep eGFR decline, whereas AGEs showed association with progression of kidney disease (steep decline of eGFR and progression of albuminuria) and incident MACE. Additionally, MG-H1 was associated with the progression of albuminuria and moderately associated with incident MACE. Overall, these findings suggest that protein glycation products are important risk factors for target organ damage in type 1 diabetes. These data also provide further support to investigate the causal role of serum protein glycation in the progression of diabetes complications.

### Electronic supplementary material

Below is the link to the electronic supplementary material.

Supplementary Material 1.

## Data Availability

No datasets were generated or analysed during the current study.
